# Surgical fixation of distal ulna neck and head fractures

**DOI:** 10.1007/s00064-023-00835-5

**Published:** 2023-11-09

**Authors:** LX van Rossenberg, BJM van de Wall, N Diwersi, L Scheuble, FJP Beeres, M van Heijl, S. Ferree

**Affiliations:** 1 Department of Health Sciences and Medicine, University of Lucerne, Luzern, Switzerland; 2 Department of Surgery, Center for Hand and Wrist Surgery, Diakonessenhuis Utrecht, Utrecht, The Netherlands; 3 Department of Orthopedic Surgery and Traumatology, Luzern Kantonspital, Luzern, Switzerland; 4 Department of Surgery, Cantonal Hospital Obwalden (KSOW), Sarnen, Switzerland; 5 Netherlands Department of Traumatology, University Medical Center Utrecht, Heidelberglaan 100, 3584 CX Utrecht, The Netherlands

**Keywords:** Distal ulna fracture, Internal fixation, Distal radioulnar joint, Early active motion, Surgical outcome, Distale Ulnafraktur, Interne Fixation, Distales Radioulnargelenk, Frühfunktionelle Nachbehandlung, Operatives Ergebnis

## Abstract

**Objectives:**

Distal ulna plate fixation for ulnar neck and head fractures (excluding ulnar styloid fractures) aims to anatomically reduce the distal ulna fracture (DUF) by open reduction and internal fixation, while obtaining a stable construct allowing functional rehabilitation without need for cast immobilization.

**Indications:**

Severe displacement, angulation or translation, as well as unstable or intra-articular fractures. Furthermore, multiple trauma or young patients in need of quick functional rehabilitation.

**Contraindications:**

Inability to surgically address concomitant ipsilateral extremity fractures, thus, limiting early active rehabilitation. Stable, nondisplaced fractures. Need for bridging plate or external fixator of distal radiocarpal joint.

**Surgical technique:**

An ulnar approach, with a straight incision between the extensor and flexor carpi ulnaris. Preservation of the dorsal branch of the ulnar nerve. Reduction and plate fixation with avoidance of plate impingement in the articular zone.

**Postoperative management:**

Postoperatively, an elastic bandage is applied for the first 24–48 h. In isolated DUF with stable fixation, a postoperative splint is often unnecessary and should be avoided. For the first four weeks, only light weightbearing of everyday activities is allowed to protect the osteosynthesis. Thereafter, heavier weightbearing and activities are allowed and can be increased as tolerated.

**Results:**

The best available evidence likely shows that for younger patients with a DUF, with or without concomitant distal radius fractures, open reduction and internal fixation can be safely achieved with good functional outcome and acceptable union and complication rates as long as proper technique is ensured.

## Abstract (de)

**Zielsetzung:**

Die Versorgung von Frakturen des Ulnahalses und -kopfes (mit Ausnahme von Frakturen des Processus styloideus ulnae) mittels Plattenosteosynthese verfolgt das Ziel einer anatomischen Reposition durch offene Reposition und interne Fixation. Hierdurch sollte ein stabiles Konstrukt erreicht werden, um eine funktionelle Nachbehandlung ohne die Notwendigkeit einer Ruhigstellung im Gips zu ermöglichen.

**Indikationen:**

Schwere Dislokation, Angulation oder Translation sowie instabile oder intraartikuläre Frakturen. Des Weiteren Mehrfachtraumata oder junge Patienten, die eine schnelle funktionelle Rehabilitation benötigen.

**Kontraindikationen:**

Vorliegen von nicht operablen, ipsilateralen Extremitätenfrakturen, die eine frühfunktionelle Nachbehandlung unmöglich machen. Stabile, undislozierte Frakturen. Notwendigkeit eines internen oder externen Fixateurs zur Ruhigstellung des radiokarpalen Gelenks.

**Operationstechnik:**

Ulnarer Zugang mit gerader Inzision zwischen dem M. extensor carpi ulnaris und dem M. flexor carpi ulnaris. Schonung des distalen Astes des N. ulnaris. Anatomische Reposition und Plattenosteosynthese unter Vermeidung eines Impingements von Platte und Gelenk.

**Postoperatives Prozedere:**

Postoperativ wird für die ersten 24–48 h eine elastische Bandage angelegt. Bei isolierten distalen Ulnafrakturen mit stabiler Osteosynthese ist ein Gips postoperativ oft unnötig und sollte vermieden werden. Zum Schutz der Osteosynthese sind in den ersten vier Wochen postoperativ lediglich leichte alltägliche Belastungen erlaubt. Anschließender Belastungsaufbau nach Maßgabe der Beschwerden.

**Ergebnisse:**

Die verfügbare Evidenz zeigt, dass bei jungen Patienten mit einer distalen Ulnafraktur mit oder ohne begleitende distale Radiusfraktur eine offene Reposition und interne Fixierung mit guten funktionellen Ergebnissen und einer akzeptablen Heilungs- und Komplikationsrate sicher durchgeführt werden können, solange die operativen Standards eingehalten werden.

## Introductory remarks

Distal ulna fractures (DUF) are a frequent concomitant injury in distal radius fractures (DRF) and to a lesser extent observed as isolated injury. The mechanism of injury is most often a fall on an outstretched hand. The distal ulna comprises the ulnar styloid, ulnar head, and distal ulnar metaphysis (neck). However, a distinction is often made between ulnar styloid process (USP) fractures and ulnar head and neck fractures. Logan et al. describes ulnar head fractures as either solitary or combined with an extra-articular component of the distal ulna (e.g., ulnar styloid). Ulnar neck fractures are considered so if they are within 5 cm of the distal dome of the ulnar head [16]. Ulnar styloid process fractures seldomly occur as a solitary fracture, but are most regularly observed as concomitant injury in distal radius fractures (± 60% of cases) [19]. Most USP fractures can be managed nonsurgically without compromising functional outcome [20]. However, in case of distal radioulnar joint (DRUJ) instability, triangular fibrocartilage complex pathology, or USP non-union, surgical fixation may be required [3]. Szalay et al. demonstrated that fixation of the USP with an angle stable hook plate is a viable and successful option when surgery is indicated [12]. Therefore, this article will focus on surgical treatment of distal ulna fractures excluding fractures of the ulnar styloid process.

**Fig. 1:**
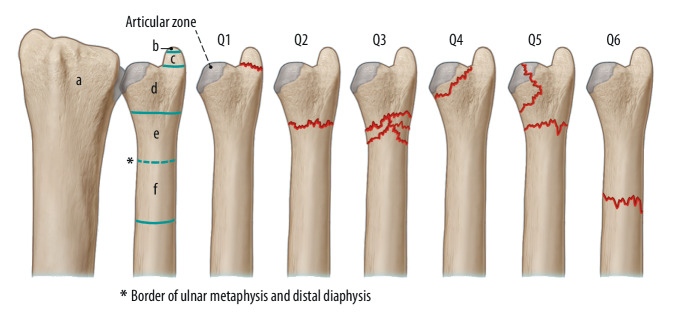
Comprehensive “Arbeitsgemeinschaft für Osteosynthesefragen” (AO) classification of distal ulnar fractures. *a* Radius, *b* Styloid tip, *c* Ulnar styloid, *d* Ulnar head, *e* Ulnar neck, *f* Distal ulnar shaft, *Q1* Ulnar styloid fracture, *Q2* Simple ulnar neck fracture, *Q3* Comminuted ulnar neck fracture, *Q4* Ulnar head fracture, *Q5* Combined ulnar head and neck fracture, *Q6* Distal ulnar shaft fracture. *Dashed blue line* Border of ulnar metaphysis and distal diaphysis, *solid blue line* Articular zone, *red line* Fracture line

For the treatment of distal ulnar fractures, excluding USP, evidence is sparse and limited to case series, retrospective studies, and only a few prospectively designed studies [6, 7, 10, 18, 19]. Distal ulna fractures are observed as a concomitant injury in distal radius fractures in approximately 5% of cases [3]. Isolated ulnar head and neck fractures comprise less than 20% of all (non USP) DUF fractures [19]. The “Arbeitsgemeinschaft für Osteosynthesefragen” (AO) has established a comprehensive, simple, and frequently used classification, although it does not predict outcome or dictate treatment decisions (Fig. 1; [1]).

For DUF, necessity of fixation can be debated and is not commonly performed. For example, in elderly patients with DUF and concomitant DRF, conservative management of the DUF with cast immobilization has proven successful after rigid fixation of the DRF [5, 15, 21]. However, fixation of DUF restores anatomical alignment and congruency of the DRUJ and allows for early mobilization. This is important as over time articular incongruity of the joints in the wrist (DRUJ, radiocarpal and midcarpal) leads to osteoarthritis in over 90% of patients [13]. Furthermore, fixation restores tension on the distal oblique bundle which in turn also adds to DRUJ alignment [2]. This also could be advantageous to prevent DRUJ instability and subsequently osteoarthritis [2, 27]. Fixation of DUF also prevents secondary problems related to ulnar and DRUJ instability after mal- or non-union of the distal ulna.

In concomitant DRF, fixation of the radius may restore DRUJ congruency and stability by tension on the distal oblique bundle. However, this is dependent on fracture morphology related to the distal oblique bundle anatomy [15]. Therefore, several previous reports have suggested to assess DRUJ stability after DRF fixation and perform DUF fixation in cases of instability [5, 10, 15]. Furthermore, DUF fixation could be advantageous to aid in stability of the radius open reduction and internal fixation (ORIF) and allows early active motion. Other indications for DUF fixation mentioned in literature are fracture angulation of ≥ 10°, ≥ 3 mm of ulnar shortening, or translation ≥ 1/3 of the diaphysis, instability of the distal ulna head/neck or fracture fragment motion with passive forearm motion and lastly articular displacement [6, 23, 26, 28]. However, it should be noted that these suggested indications are based on expert opinion rather than scientific evidence.

The purpose of this paper is the description of the surgical technique for this delicate procedure.

## Surgical principle and objective

Plate osteosynthesis of the distal ulna to anatomically reduce the distal ulna fracture by open reduction and internal fixation to obtain a stable construct allowing functional rehabilitation without need for cast immobilization.

## Advantages

- Increased stability compared to conservative treatment in concomitant distal radius fracture
- More precise alignment of the displaced fracture
- No need for cast treatment, thus, allowing early active range of motion

## Disadvantages

- Increased risk of fracture related infection
- Risk of damage to dorsal cutaneous branch of the ulnar nerve (DBUN)
- Risk impingement during forearm rotation
- Risk of extensor carpi ulnaris (ECU) irritation due to osteosynthesis
- Risk of chronic luxation of ECU tendon by extensor retinaculum damage
- Risk of secondary removal of osteosyntheses and adjoining complication risks
- Risk of damage to the ulnar vessels and ulnar nerve while drilling at palmar side

## Indications

The decision to perform ORIF in DUF should be based on more than fracture pattern alone and should account for specific demands of the patient. The following indications provide a guideline:

- Displaced and/or unstable fractures (instability of ulnar head/neck or motion of fracture fragments during passive motion) with or without concomitant distal radius fracture which requires ORIF
- Incongruency in DRUJ (both with or without concomitant distal radius fracture)
- Open fractures (excluding acceptable reduced stable fractures after wound debridement)
- Patient with bilateral extremity fracture and need for early active rehabilitation (multiple trauma)
- High demands of the patients with regard to the level of activity

## Contraindications

- Inability to surgically address concomitant ipsilateral extremity fractures, thus, limiting early active rehabilitation
- Stable, nondisplaced fractures
- Severe osteoporosis of the ulnar head in which screws cannot hold
- Need for bridging plate or external fixator of distal radiocarpal joint

## Patient information

- General patient information
- Need for future plate removal due to irritation
- Risk of damage to DBUN

## Preoperative workup

- Preoperative radiologic evaluation (including standard X‑rays of the injured and unaffected side as well as computed tomography scan) to determine fracture pattern, stability and DRUJ involvement, ulnar variance and DRUJ congruency
- Preoperative admission of 2 g cefazoline intravenously within 60 min prior to incision

## Instruments and implants

- Standard surgical instruments for soft tissue procedures and osteosynthesis
- Small drill with 1.8 mm drill head and 2.4 mm cortical and variable angle locking screws
- K‑wires of different sizes for temporary fixation if necessary
- Variable angle locking plate size 1.5 to 2.0 mm depending on patient size

## Anesthesia and positioning

**Fig. 2:**
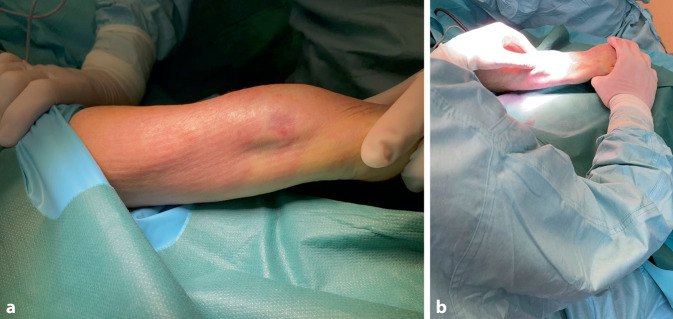
**a** The patient is placed in supine position with the arm fully abducted and resting on a mobile arm table. The wrist is fully pronated. Optionally the wrist can be slightly elevated to create a better angle of approach (example of elevating technique in Fig. 3). **b** The surgeon is working in a seated position

- General anesthesia or axillary plexus block
- Supine position of the patient, arm table and upper arm tourniquet
- Arm abducted, wrist pronated and supported by elevating structure (± 20°; Fig. 2a)

## Surgical technique

(Figs. 3, 4, 5, 6, 7, 8, 9 and 10)

**Fig. 3:**
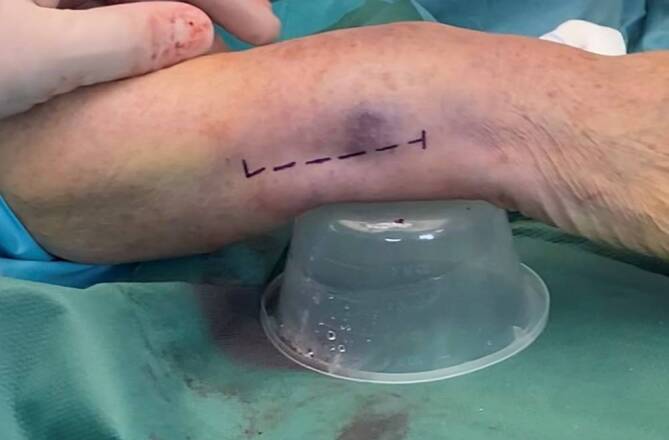
For optimal surgical access, the ulnar approach is applied. Before incision the ulnar styloid is palpated (distal transversal line), the ulnar ridge of the ulna is palpated 5 cm proximal (proximal transversal line). A straight, longitudinal incision between the extensor and flexor carpi ulnaris is made (*dashed line*), approximately 5 cm in length, starting at the level of the ulnar styloid process (USP). In order to protect the dorsal branch of the ulnar nerve (DBUN), care is taken to limit the incisional depth to the dermis, especially in the distal part of the incision

**Fig. 4:**
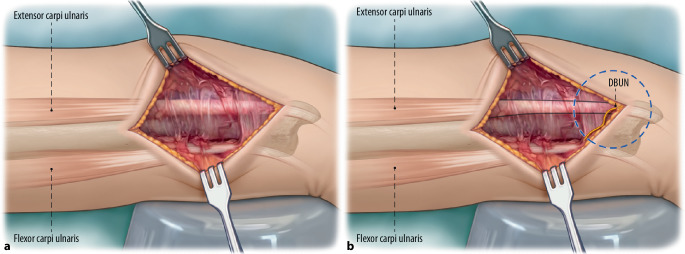
**a** Skin retractors are used to display the surgical site. The dorsal branch of the ulnar nerve must be identified. The dorsal branch of the ulnar nerved (DBUN) emerges at the dorsal border of the flexor carpi ulnaris on average 5 cm proximal to the pisiform. It then runs subcutaneously and crosses volar over the extensor carpi ulnaris (ECU) [4, 9]. Further division of the subcutaneous tissue is therefore performed with spreading scissors until the fascia is encountered. **b** *Blue dashed circle *indicates the area where the DBUN can often be encountered*. Yellow line *Schematic trajectory of DBUN. *Black lines *Indicate the volar (*lower line*) and dorsal (*upper line*) borders of the ECU tendon

**Fig. 5:**
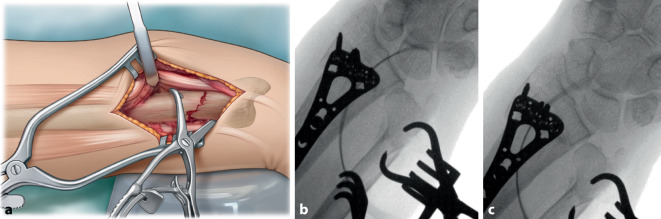
**a** The fracture is visualized and reduced using clamps (Weber). Depending on fracture pattern, temporary fixation of the distal fragment to the distal radius with a Kirschner (K)-wire can be beneficial. This may have the advantage in case the Weber clamps hinder plate positioning. **b** To ensure anatomic reduction, fluoroscopy is performed. **c** Distal radioulnar joint (DRUJ) congruency is assessed

**Fig. 6:**
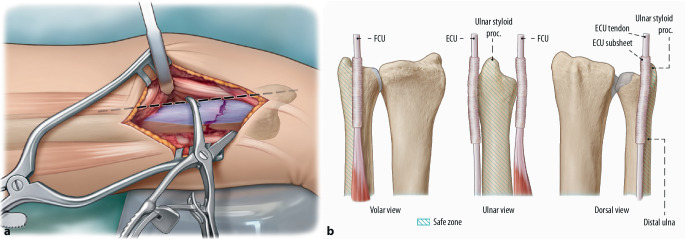
**a** Plate positioning must be performed meticulously to prevent ECU impingement and rotational limitation. Determining the safe zone and using adequate size of distal ulna plate (1.5 or 2.0 mm locking compression plate [LCP]). The safe zone comprises the ulnar border and a small area volar of the ulnar border. The plate should be placed at the level of the USP or volar, within the 90° of non-articulating area. In fractures requiring extreme distal placement a maximum plate width of 20 mm and volar placement avoids impingement. In case dorsal placement is required a similar plate placed immediately volar to the ECU tendon is possible. However, extra care should be taken regarding the subsheet of the ECU tendon and patients must informed of greater risk of complications. *Dashed black line *Ulnar border in line with the ulnar styloid process. *Blue area *Indicates safe zone for plate fixation. If the *dashed black line *is considered 0°, the safe zone ranges from approximately 0 to 30° volar [9]. **b** Schematic figure indicating the safe zone for plating of the distal ulna in volar, ulnar and dorsal view. *FCU* Flexor carpi ulnaris. *Blue stripes* Indicates safe zone

**Fig. 7:**
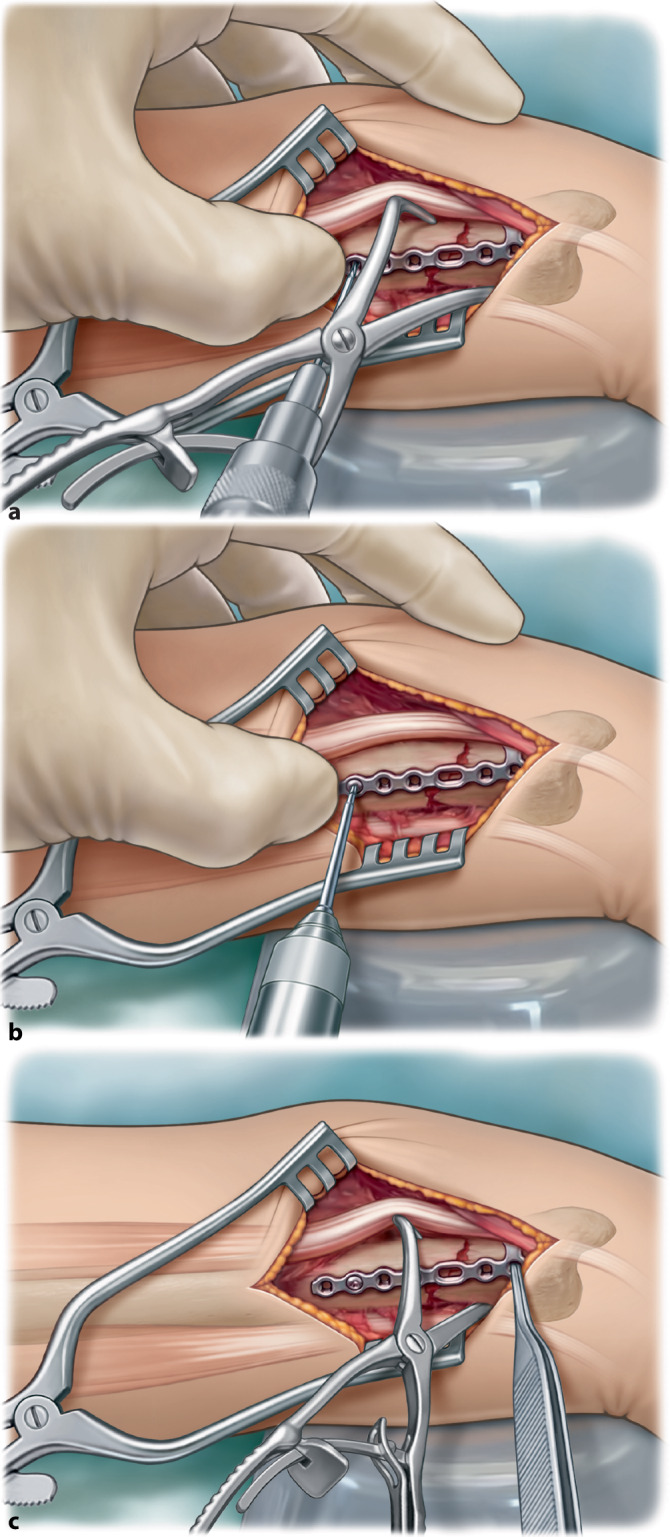
**a** For comminuted fractures the plate is contoured to ensure an as low profile fit as possible. Consecutively proximal fixation of the plate is performed first with a 1.8 mm drill and **b** 2.4 mm cortical screw. **c** This allows slight adjustment (indicated with pincers) of the plate distal by rotation of the plate around the proximal screw

**Fig. 8:**
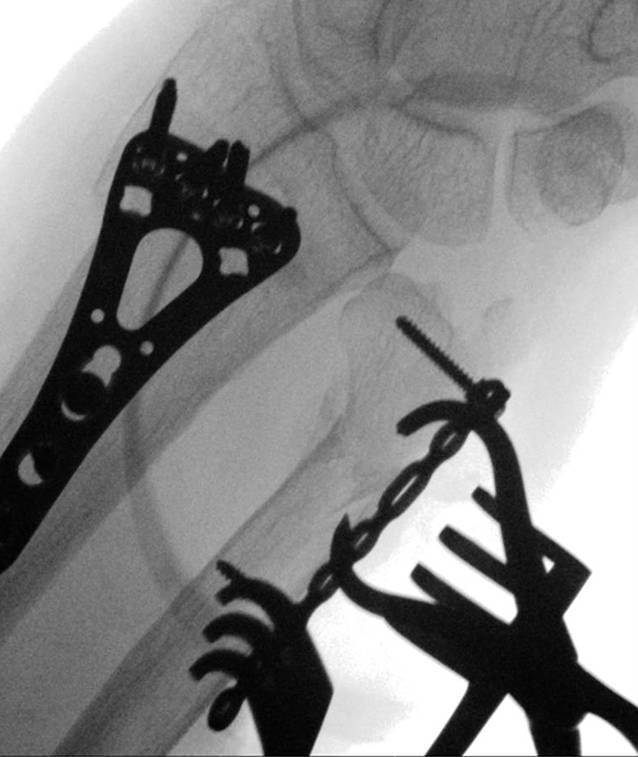
After proximal fixation, the distal fracture part is fixated with a 2.4 mm variable angle locking screw. Ulnar height is assessed with fluoroscopy to ensure anatomic reduction remained adequate during fixation. In case of doubt of adequate reduction intra-operatively, USP height and subsequently rotation can be assessed by comparing to the preoperative X‑ray images of the unaffected extremity in similar pronation–supination position

**Fig. 9:**
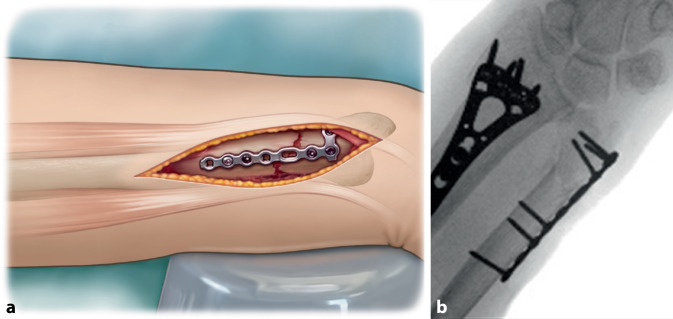
**a** After examination of correct plate positioning and confirmation with fluoroscopy, the plate is fully fixated. Proximal fixation is performed with two cortical screws and one variable angle (VA) locking screw. Distal fixation is performed with four VA locking screws. It is paramount that the screws do not protrude the ulnar head into the distal radioulnar joint. **b** Fluoroscopy imaging is performed to assess the position of the fully fixated plate. *Note: *In this case the surgeon used a T-plate which was adjusted manually to ensure proper positioning in the safe zone. See volar side of distal end of plate see in **a**

**Fig. 10:**
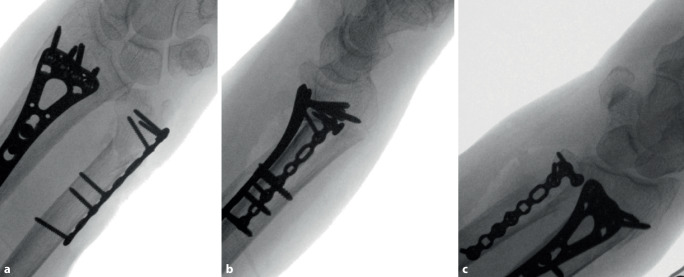
Next a full range of motion exam of the wrist is performed with fluoroscopy imaging to assess any limitations that might have occurred. The surgeon assesses potential impingement during the exam. In case of limitations, plate positioning must be reconsidered. Intraoperative images are made in **a** pronated, **b** neutral and **c** supinated position

Before wound closure, DRUJ stability is assessed using the manual shuck examination maneuver [11]. Comparison with the uninjured side can be helpful to adequately assess instability. Any residual DRUJ instability can now be addressed depending on the injury pattern. Many specific techniques to achieve this are available; however, USP fracture fixation when present or fixation of the distal ulna to the distal radius in stable rotational position with a K-wire are most frequently used. In this case, DRUJ was stable. The wound is sutured intracutaneously.

## Special surgical considerations

- X‑rays preoperative, during surgery and postoperative
- Excessive soft tissue retraction in distal part of incision should be avoided to prevent damage to DBUN
- It should be confirmed that plate placement does not cause impingement or ECU tendon irritation
- The ulnar variance should be assessed comminuted fractures by comparing to uninjured side
- DRUJ stability and congruency should be controlled by manual shuck examination maneuver pronated, neutral and supinated, neutral and supination

## Postoperative management

Postoperatively, an elastic bandage is applied for the first 24–48 h. In isolated DUF with stable fixation, a postoperative splint is often not necessary and should be avoided. The goal is to ascertain active range of motion early after surgery, thus, fixation should strive to provide enough stability to allow this.

Alternatively, the wrist is placed in a short lower arm splint for pain control and soft tissue healing for 2–4 weeks. This could be indicated in cases with concomitant distal radius fracture ORIF, osteoporotic bone and/or uncertainty of fracture stabilization.

A special indication for postoperative casting could be persistent DRUJ instability after DUF fixation. In this situation the primary choice of treatment is upper arm casting in a stable position for 4–6 weeks, to maximize limitation of pro- and supination.

For the first 4 weeks, only light weightbearing (weight < 2 kg) of everyday activities is allowed to protect the osteosynthesis. Thereafter, heavier weightbearing and activities are allowed and can be gradually increased as tolerated.

Postoperative outpatient clinic evaluation is performed with standard anteroposterior (AP) and lateral radiographs. As a general guideline this could be done at: 2 weeks to assess early surgical failure and any revision at this stage is possible; 6 weeks to assess early signs of consolidation and osteosynthesis integrity (i.e., early signs of bone healing issues like delayed union/non-union could be visible at this point as osteolysis around screws or hardware loosening); 3 months to assess full consolidation of the fracture. At this stage, range of motion can also be assessed and especially forearm rotation should be determined.

Plate removal is indicated in patients with complaints at 6 months or later.

## Errors, hazards, complications

- DBUN damage resulting in neuroma or hypesthesia in the ulnar side of the hand
- Protrusion of screws into the DRUJ
- Nonanatomic reduction leading to DRUJ incongruency and limitations to forearm rotation
- Over or under reducing ulnar height leading to ulnar carpal abutment or DRUJ-related issues
- Relative positive ulnar variance (with ulnar carpal abutment with result) can occur with anatomical reduction and fixation of DUF in concomitant DRF with loss of radial height
- Plate position outside safe zone, causing impingement or ECU irritation
- ECU subsheet damage resulting in instability or luxation of ECU tendon during wrist rotation
- Persistent DRUJ instability after DUF fixation
- Secondary dislocation due to hardware failure

## Results

### Case report

**Fig. 11:**
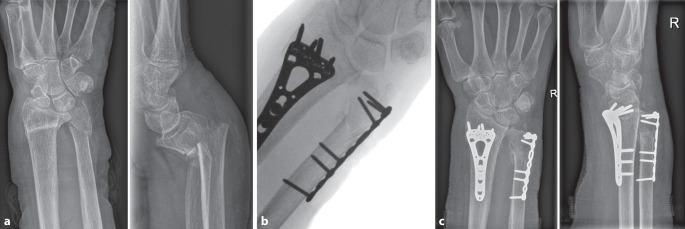
Pre-, intra-, and post-operative images of patient with distal ulnar fracture and concomitant distal radius fracture. **a** Pre-operative X‑ray images in the anteroposterior (AP) and lateral direction. **b** Intraoperative fluoroscopy image of fully fixated distal radius and distal ulna fractures. **c** Post-operative X‑ray images in AP and lateral direction with osteosynthesis in situ

An 81-year-old woman was admitted to the emergency room (ER) after a fall on her outstretched right hand. During physical examination swelling, functional limitation, dislocation and pain were observed. X‑ray imaging showed a volar angulated distal radius and subcapital ulna fracture (Fig. 11a). After unsuccessful reposition and secondary dislocation the patient was advised to undergo surgery for both distal radius and distal ulna fractures via open reduction internal fixation. Intraoperative fluoroscopy images showed anatomic reduction of the distal radius and ulna fractures (Fig. 11b). Follow-up X‑rays showed adequate fracture healing with maintained radial height, angulation and DRUJ congruency (Fig. 11c).

### Outcome literature

Several studies have analyzed outcome of operative techniques on the distal ulna. Ring et al. retrospectively analyzed the outcome of unstable DUF with concomitant DRF after minicondylar blade plate [23]. Twenty-four patients with 24 months follow-up were assessed for functional outcome and union rate. They found healing with good radiographical alignment, function (Gartland and Werley system 4 points) and 1 secondary surgery due to non-union after a grade 3 open fracture. A removal rate of 29% was found.

Han et al. reviewed results of their locked compression plating in unstable DUF with concomitant DRF. Seventeen patients with a follow-up of 15 months were included in this retrospective review. All patients went on to union and had good to excellent Sarmiento’s modified wrist scores [8].

Dennison retrospectively reviewed 5 patients with unstable DUF in concomitant DRF, who underwent ORIF [6]. All patients went on to union, had good to excellent alignment and motion, and nearly symmetric grip strength.

Ozkan et al. retrospectively identified 277 patients with an ulnar neck fracture associated with a DRF [22]. The purpose of their study was to identify factors associated with unplanned secondary surgery. Fifty-six (20%) patients received operative intervention for the DUF of which 6 (11%) needed secondary surgery versus only 1 (0.5%) in the non-operative group. Factors associated with unplanned surgery were the following: younger age, open and multifragment fractures, and initial surgical treatment of the ulnar neck fracture.

Sato et al. retrospectively reviewed all patients aged over 60 years who received conservative treatment for DUF with concomitant DRF [25]. All fractures united and functional outcome by modified Gartland and Werley scores were excellent in all but one patient. The disability of arm shoulder and hand (DASH) score was 4.2 which is considered normal.

Ruchelsman et al. performed Darrach resection of the distal ulna in fractures deemed unreconstructable [24]. They hypothesized that when anatomic restoration and stable fixation was not possible that resection would yield satisfactory results. Eleven patients with concomitant DUF underwent a Darrach procedure. At a mean of 42 months follow-up, the modified Gartland and Werley scores were 7 excellent and 4 good. No patients had distal ulna instability and none required secondary surgery.

Five studies compared outcome of fixation of DUF as a concomitant injury of a DRF versus non-operative treatment for the ulna [5, 7, 14, 17, 18]. Four studies were retrospective in design and only one had a prospective design [5]. The average age in all studies was above 50 years old, with the highest average age of 82 years old in the study by Lutsky et al. Kurozumi et al. and Cha et al. analyzed functional outcome with the DASH scores and found no difference between surgically and non-operatively treated patients [5, 14]. The patient-rated wrist evaluation (PRWE) was used by Moloney et al. and Glogovac et al, whereby Glogovac et al. did not find a statistically difference between the two treatment modalities [7]. Glogovac et al. also analyzed the outcome of Darrach resection. They found no statistical difference between this procedure and operative and non-operative treatment. However, the Darrach group (*n* = 5) had a PRWE score of 70, indicating severe functional disability. This was compared with a PRWE of 49 for non-operatively and 28 for operatively treated patients [7].

Moloney et al., who also performed a subanalysis of isolated DUF, found worse PRWE scores for operated DUF patients [18]. Patient rated wrist evaluation scores of 27.5 (standard deviation [SD] 36) were found for operated DUF patients compared with 7.75 (SD 22) for the non-operative group (*p* = 0.01) The isolated DUF group had a PRWE score of 7 (SD 19) versus 18 (SD 41) for the DUF with concomitant DRF. For both the isolated and concomitant DRF group, the PRWE was worse in the operated group. This study also examined the association of osteoarthritis, found radiographic signs in 22 DRUJ (33%) and this was associated with worse PRWE scores.

Range of motion was examined in four studies. Kurozumi et al. found a 30° decreased arc of dorsipalmar flexion in operated DUF patients compared with non-operative group (129 vs 158, *p* = 0.01) [14]. The other studies found no difference in range of motion.

With regard to bony union, no statistically differences were found in any of the studies. However, sample size and low prevalence of non-union may have led to a type II error. Therefore, no reliable conclusion can be drawn for these data. When all studies are combined, a non-union rate of 3.3% for operated DUF and 0.5% for non-operative patients is calculated.

Outcomes reported in the literature should be considered with care. In current practice, decision of best approach, positioning of the plate (dorsal, dorsoulnar, ulnar or palmar), and indications for surgery often differ and still pose a challenge in the treatment of distal ulnar fractures.

## Declarations

For this article no studies with human participants or animals were performed by any of the authors. All studies mentioned were in accordance with the ethical standards indicated in each case.
